# Continuous irrigation and suction with a triple-cavity drainage tube in combination with sequential somatostatin–somatotropin administration for the management of postoperative high-output enterocutaneous fistulas: Three case reports and literature review

**DOI:** 10.1097/MD.0000000000018010

**Published:** 2019-11-15

**Authors:** Xiangheng Kong, Yuning Cao, Daogui Yang, Xiangyang Zhang

**Affiliations:** a Department of Gastrointestinal Surgery; b Department of Digestion, Liaocheng People's Hospital, Liaocheng, Shandong Provence; c Department of General Surgery, Wanshan Branch of Xiangyang Central Hospital, Xiangyang, Hubei Provence, China.

**Keywords:** enterocutaneous fistula, high-output, negative pressure, somatostatin, somatotropin

## Abstract

**Introduction:**

Enterocutaneous fistula is considered one of the most serious complications in general surgery and is associated with high morbidity and mortality. Although various treatments are reported to have varying success, high-output enterocutaneous fistulas (output over 500 ml/day) continue to be associated with high mortality, and few papers on this topic exist in the literature. The aim of this study is to describe an effective multidisciplinary treatment method for postoperative high-output enterocutaneous fistula and discuss the clinical development of the therapeutic strategy.

**Patient concerns:**

Three patients suffered high-output enterocutaneous fistulas, in which case 1 presented with duodenal fistula, case 2 with ileal fistula, and case 3 with small bowel fistula.

**Diagnosis:**

: All 3 cases were diagnosed with high-output enterocutaneous fistulas by drainage of intestinal contents.

**Interventions:**

With the exception of routine treatment including fluid resuscitation, correction of the electrolyte balance, control of infection, and optimal nutrition, all the cases accepted continuous irrigation and suction with triple-cavity drainage tubes in combination with sequential somatostatin–somatotropin administration were given. With regard to establishing effective drainage, the triple-cavity tube placement was performed by insertion through the initial drainage channel in case 1, percutaneous puncture with dilation by graduated dilators in case 2, and tract reconstruction in case 3. The technical details of the approach are described and clinical characteristics including fistula location, defect size, output volume, approach of triple-cavity tube placement, length of fistula tract, somatostatin and somatotropin administration time, and fistula healing time were recorded and compared. In addition, other various techniques reported in the literature are reviewed and discussed.

**Outcomes:**

All the patients were cured by the multidisciplinary treatments and were followed up without fistula recurrence and other relevant complications at 1 week, 1 month, and 3 months after the treatments.

**Conclusion:**

The strategy involving continuous irrigation and suction with a triple-cavity drainage tube in combination with sequential somatostatin–somatotropin administration may be a safe and effective alternative treatment for postoperative high-output enterocutaneous fistula and a more practical method that is easy to execute to manage this problem. Long-term studies, involving more patients, are still necessary to confirm this suggestion.

## 1 Introduction

An enterocutaneous fistula is defined as an abnormal communication between the intra-abdominal gastrointestinal tract and the skin^[1]^ and can arise as a complication of injury from intra-abdominal surgery, malignancy, inflammatory bowel disease, or postradiation therapy for malignancy or as a result of a distal obstruction. Moreover, an enterocutaneous fistula is traditionally considered as one of the most feared complications in general surgery. Furthermore, it is associated with high morbidity and mortality, and markedly impairs patients’ quality of life.^[2,3]^ Although mortality rates have decreased significantly in the last few decades from as high as 40–65% to 5.3–21.3%, high-output fistulas (over 500 ml/day)^[4]^ continue to have a mortality rate of approximately 35%.^[5,6]^ In various studies, the presence of a high-output fistula has been found to be a poor prognostic indicator.^[7,8]^

For the management of high-output fistulas, several treatments, such as endoscopic stenting,^[9]^ percutaneous obliteration using an occluding coiled embolus and fibrin sealant,^[10]^ percutaneous transhepatic biliary/duodenal drainage,^[11]^ surgical biliogastric diversion,^[12]^ rectus abdominis muscle flap repair,^[13]^ and pedicle ileal flap repair,^[14]^ have been described sporadically in case reports. Although various treatments with varying success have been described, no single modality can be alleged to be superior to any other because of the small number of cases reported and the absence of relevant randomized controlled trials.

Due to severe edema and friable tissues at the leakage site and dense postoperative adhesions, surgical treatment should not be considered a suitable procedure. The presence of friability and tissue adhesion requires a damage control approach that avoids further damage to the bowel lesions by surgery and allows the intestinal output to exit the abdomen in an easy and direct manner to prevent contamination of the peritoneal cavity. Minimally invasive approaches to interventional management usually serve this purpose. We present a new and alternative therapeutic strategy for conservative treatment with a minimally invasive approach involving continuous irrigation and suction with a triple-cavity drainage tube in combination with sequential somatostatin–somatotropin administration for high-output enterocutaneous fistulas and discuss the clinical development of the therapeutic strategy on the basis of our treatment experience. We also perform a literature review of the various techniques used in the treatment of enterocutaneous fistulas.

## 2 Case presentation

### 2.1 Case 1

**Figure 1:**
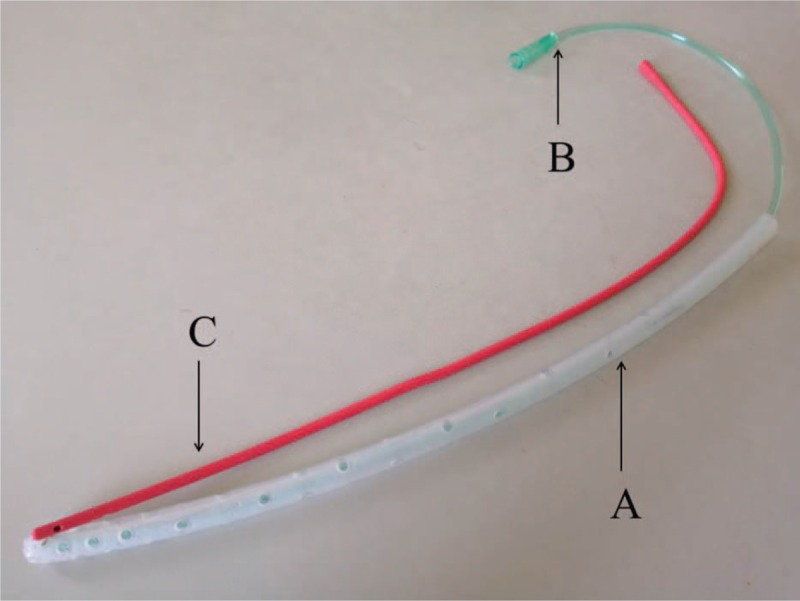
The homemade triple-cavity unit consisted of 3 different tubes: a large-bore silicone tube as an outer sleeve (A) with multiple side holes and 2 fine-bore tubes, including 1 for suction (B) inside the outer sleeve and another for irrigation (C) outside the outer sleeve. The irrigation tube is fixed to the outer sleeve.

A 27-year-old man with a duodenal injury from a traffic accident was treated with simple closure for duodenal rupture, retrograde placement of a drainage tube through the jejunum for duodenal decompression, and preventive jejunostomy for enteral nutrition (EN) in another hospital. Seven days after the operation, he was found with bile leaking from the drainage tube, which gradually developed into an intra-abdominal abscess; septic episodes occurred within the next 3 days. Then, he was transferred to the intensive care unit of our hospital due to persistent sepsis. On admission, bile leakage from the drainage tube exceeded 800 ml in the first 24 hours. For sepsis control, a broader spectrum antibiotic was administered. An acid inhibitor drug, omeprazole, and somatostatin analog, octreotide, were administered to reduce the output. Total parenteral nutrition (TPN) was given due to poor intestinal function. An abdominal computed tomography (CT) exam and fistulography revealed a 2-cm defect on the junction of the descending part and horizontal part of the duodenum and a large surrounding fluid collection. Because of the high risk of a second operation, a damage control strategy should be considered in these cases. Thus, conservative treatment was selected. To facilitate effective drainage, active suction drainage was needed instead of the previous passive drainage method. Thus, a homemade triple-cavity tube (Fig. 1) with functions of continuous irrigation and suction, which was designed with an insertion length of approximately 14 cm as determined by measuring the previous drainage tube of the intra-abdominal part on fistulography, was immediately inserted in the direction of the duodenal defect along the initial drainage channel when the previous drainage tube was removed. Then, the location of the triple-cavity tube was identified and slightly adjusted near the duodenal defect under fistulography. Negative pressure was applied at approximately −100 mm Hg. With the patient's stabilization 7 days later, the fistula tract formed gradually and was identified by repeated fistulography. Due to intestinal function recovery, octreotide was reduced and eventually stopped, and EN was administered through the jejunostomy tube. After EN was well tolerated at 40 kcal/kg/day of energy and completely replaced TPN, somatotropin (recombinant human growth hormone) was administered at a dosage of 8 U/day. An interesting phenomenon was that the triple-cavity tube was frequently plugged by exudative fibrous tissue approximately 14 days after recombinant human growth hormone administration and thus needed to be rinsed at least twice a day. To promote healing, the tube was pulled out approximately 2 cm to facilitate fistula tract closure. Eighteen days after recombinant human growth hormone administration, the duodenal fistula had healed completely, and the patient resumed oral intake. The patient was followed without fistula recurrence and other relevant complications at 1 week, 1 month, and 3 months.

### 2.2 Case 2

A 32-year-old woman with a diagnosis of a hydatidiform mole at a gestational age of 16 weeks underwent uterine curettage in another hospital. On the 6th postoperative day, the patient presented with fever and aggravated abdominal pain. Then, she was transferred to our department for severe peritonitis and underwent an emergency exploratory laparotomy. The intraoperative findings included massive intra-abdominal intestinal contents and pus, severe edematous and adhesive intestines, a 1-cm defect on the intestinal wall located approximately 30 cm from the caecum and a perforation on the uterus. Simple repair of the intestinal defect and uterus and peritoneal irrigation were performed during the operation. Unfortunately, the patient suffered lower abdominal pain and fever again on the 7th day after the operation. Blood examination showed increased leukocytes and neutrophils. Abdominal CT showed a lower intra-abdominal abscess of approximately 10 cm in diameter and surrounding disordered bowel structures. Under the guidance of color Doppler ultrasound, diagnostic abdominal puncture was carried out, which yielded puncture fluid containing intestinal contents. Intestinal suture line leakage was diagnosed. Because of severe edematous and adhesive intestines, conservative treatment was selected. Under ultrasound guidance, an appropriate path that would allow subsequent dilatation was selected. Under local anesthesia, access to the abscess cavity was maintained achieved using a guidewire, over which the catheter tract was dilated to 30F using graduated dilators of a percutaneous nephroscope. Then, the triple-cavity tube was placed through the tract and advanced to the bottom of the abscess cavity. Leakage along the suture line with an approximately 2-cm defect was identified by fistulography. Continuous irrigation and suction with negative pressure at approximately −100 mm Hg was initiated, and the net output exceeded 600 ml/day for the first few days. A broader spectrum antibiotic was administered for sepsis control, and octreotide was given to decrease the output. TPN was given because of poor intestinal function. Fistulography was performed again 7 days later, which revealed fistula tract formation. Because of anus exhaust, oral feeding with starchy foods, complete protein foods, and fruit juice was encouraged, and recombinant human growth hormone was administered at a dosage of 8 U/day instead of octreotide. Although the output increased for a few days, the drainage was unobstructed, and anal defecation always occurred. Due to the antagonism between somatostatin and somatotropin, oral loperamide hydrochloride capsules but not octreotide were given to reduce intestinal secretions. Sixteen days after recombinant human growth hormone administration, the fistula tract had healed completely. Follow-up at 1 week, 1 month, and 3 months were performed without fistula recurrence and other relevant complications.

### 2.3 Case 3

**Figure 2:**
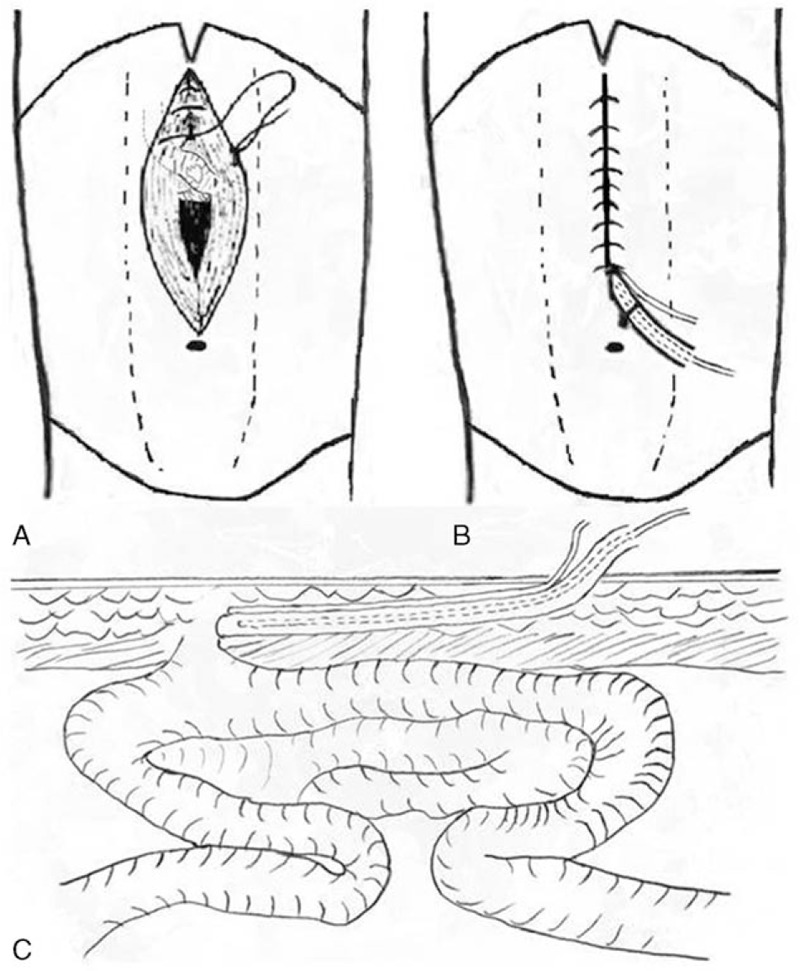
A schematic diagram of fistula tract reconstruction. (A) A defect on the small intestine exposed to the air and the re-sutured skin with fistula tract reconstruction. (B) Front view of embedding the triple-cavity tube in subcutaneous tissues. (C) Section view of embedding the tube in subcutaneous tissues.

A 53-year-old man presented with subcutaneous hydrops and abdominal distension on the 3rd postoperative day after exploratory laparotomy for intestinal obstruction due to an abdominal cocoon in another hospital. He was transferred to our department on the 7th day because of incision exudation similar to intestinal contents and progressive abdominal distension. Abdominal CT showed dehiscence of a fraction of the linea alba and subcutaneous hydrops. To verify the characteristics of subcutaneous hydrops, a skin suture was removed, and the intestinal contents flowed out of the subcutaneous cavity. When several other sutures were removed sequentially, an approximately 1-cm defect on the small intestine was identified with exposure to the air. Re-suturing of the intestinal defect and linea alba was initially attempted but failed because of the friability of the bowel wall and the severe adhesions surrounding the incision. However, severe adhesions also prevented the spread of infection. Thus, conservative treatment was the only option. Under local anesthesia, a new fistula tract measuring approximately 6 cm was constructed with embedding of the triple-cavity tube in subcutaneous tissues (Fig. 2). Continuous negative pressure at −80 mm Hg was applied, and the net output exceeded 500 ml/day in the first few days. Because of the mild infection, an antibiotic was administered according to a bacterial culture sensitivity test. Octreotide and TPN were administered until controlled fistula tract formation was identified by fistulography 7 days later. After anus exhaust, an elemental diet was given, and recombinant human growth hormone was administered at a dosage of 8 U/day instead of octreotide. Oral loperamide hydrochloride capsules were administered to reduce the intestinal output. However, no apparent signs of tract growth were found when recombinant human growth hormone was administered for 3 weeks. Thus, to avoid the side effects of long-term use, recombinant human growth hormone administration was stopped. The fistula finally healed on the 48th day after tract construction. No fistula recurrence and other relevant complication appeared during follow-up at 1 week, 1 month, and 3 months.

In addition, clinical characteristics of all the 3 cases including fistula location, defect size, output volume, approach of triple-cavity tube placement, length of fistula tract, somatostatin and somatotropin administration time, and fistula healing time were recorded (Table 1).

**Table 1:**
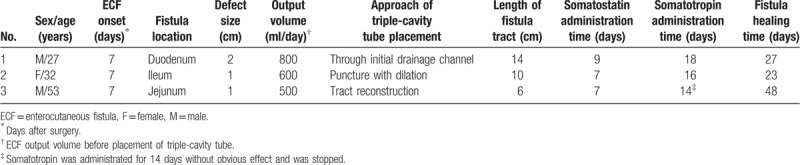
Characteristics of patients with ECF treated by continuous irrigation and suction with somatostatin–somatotropin sequential administration.

## 3 Discussion

The management of enterocutaneous fistulas is challenging and associated with high morbidity and mortality,^[5]^ which are primarily related to complications such as fluid loss, electrolyte imbalance, malnutrition, and sepsis.^[15]^ Therefore, recognizing and treating these complications are very important for reducing morbidity and mortality from enterocutaneous fistulas.^[4]^ Once an enterocutaneous fistula is recognized, emphasis on patient stabilization has been a general first step in the management, which includes fluid resuscitation, correction of the electrolyte balance, control of infection and sepsis, optimal nutrition, and treatment of acidosis.^[16]^

After patient stabilization, management of the enterocutaneous fistula is the next vital step. Various treatments have been described, with success rates of fistula closure ranging from 25% to 75%,^[17]^ such as abscess drainage, transhepatic biliary drainage,^[17]^ T-tube duodenocholangiostomy,^[18]^ fistuloscopy,^[19,20]^ fistula obliteration by cyanoacrylate or prolamine,^[21]^ percutaneous transhepatic biliary drainage and balloon occlusion,^[22]^ or more recently, negative pressure wound therapy.^[23]^ However, several treatments for high-output fistulas, such as endoscopic stenting,^[9]^ percutaneous obliteration using occluding coiled embolus and fibrin sealant,^[10]^ percutaneous transhepatic biliary/duodenal drainage,^[11]^ surgical biliogastric diversion,^[12]^ rectus abdominis muscle flap repair^[13]^, or pedicle ileal flap repair,^[14]^ have been described only sporadically in cases reports. Moreover, no single therapeutic modality for high-output fistulas has been confirmed to be superior to any other.

Recently, a trend towards conservative management for enterocutaneous fistulas with a policy of “wait and watch for 4–6 weeks”^[24]^ rather than surgical treatment has emerged.^[25]^ In general, surgical treatment involving resection and anastomoses should not be considered a suitable procedure due to the presence of edematous, friable and necrotic tissues adjacent to the fistula site. Patients with high-output, small bowel fistulas and a history of an open abdomen have an especially high likelihood of a recurrent fistula after surgical treatment. In a large series, the recurrence rate of enterocutaneous fistula following operative repair was found to be approximately 20.5%.^[26]^ Surgery should only be considered in cases of diffuse peritonitis, intra-abdominal hemorrhage, or major wound disruption to prevent further leakage and to promote early maturation of the fistula tract.^[27]^

Therefore, based on a damage control approach, the general principle of enterocutaneous fistula management should be to prioritize conservative treatment with or without a minimally invasive approach. All examinations and evaluations should address whether the fistula can be treated conservatively. Among the examinations reported in the literature, fistulography is the most common method, including traditional fluoroscopic and roentgenographic tests or CT imaging.^[12]^ In later years, fistuloscopy was proposed as an alternative, although this technique requires special training and tools.^[19]^ In our experience, both CT imaging and fistulography with a contrast agent should be performed to identify several points, including the location of the fistula or abscess cavity, the size of the bowel wall defect, the length of the fistula tract, a fistula with the bowel in continuity or complete disruption, and the presence of a distal obstruction. Once the fistula assessment shows a small bowel wall defect with a longer fistula tract, the bowel in continuity and the absence of obstruction, then conservative treatment with or without a minimally invasive approach should be selected.

However, Magalini et al^[23]^ proposed 3 main problems in the challenging situation of an enterocutaneous fistula: intraperitoneal leaks, which are sometimes near delicate intestinal structures and difficult to identify; consistent output; and corrosive secretions. Therefore, if an approach can enable effective and continual drainage of intestinal contents out of the abdomen, then the enterocutaneous fistula may spontaneously close.

**Figure 3:**
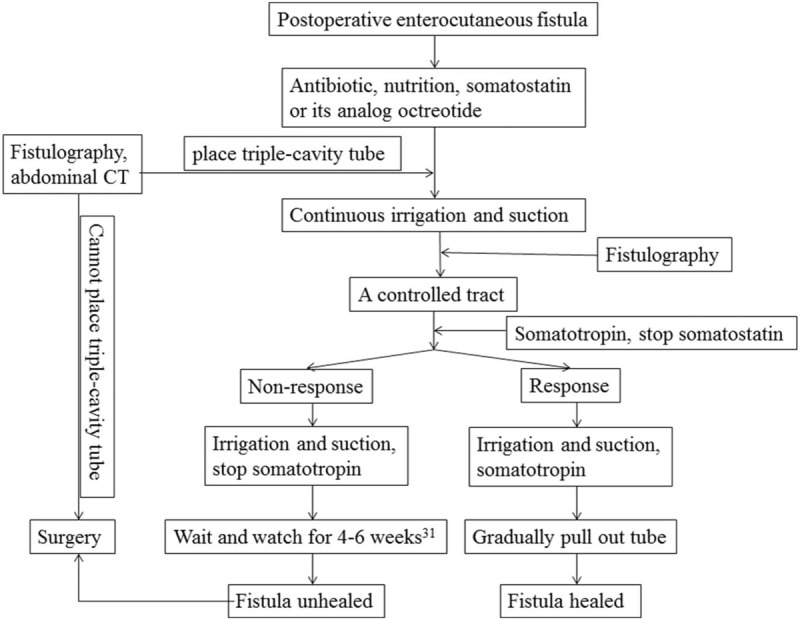
The flow diagram of postoperative enterocutaneous fistula treatment strategy.

With the aim of facilitating rapid spontaneous fistula healing, the treatment strategy involving continuous irrigation and suction with a triple-cavity drainage tube in combination with sequential somatostatin–somatotropin administration is described in this paper (Fig. 3). From our point of view, the targets of facilitating rapid spontaneous fistula closure in this therapeutic modality can be summarized into two objectives:

(1) to create a stable and controlled fistula tract and
(2) to promote healing of the fistula tract.

The former objective is the key factor in successful treatment of an enterocutaneous fistula.

To accomplish the first treatment target of creating a stable and controlled fistula tract, the main focus should be on 2 aspects: establishing more effective drainage and reducing digestive secretions. With regard to establishing effective drainage in this series, several approaches for placement of the tube, such as insertion through the initial drainage channel and percutaneous puncture with dilation by graduated dilators and tract reconstruction, were described as successful experiences. In addition, the homemade triple-cavity tube is used for effective drainage, and the outer sleeve can prevent both aspiration damage to surrounding tissues and blockage of the internal suction tube, while the inlet tube with irrigation can dilute the intestinal contents in the infected residual cavity, which can result in not only easy drainage by the suction tube facilitated by the negative pressure technique but also a sharp decrease in corrosion by digestive juices. Based on these advantages, granulation tissue grows in a relatively clean environment and facilitates rapid fistula tract formation. Furthermore, as performed in this series, placement of a triple-cavity tube is a relatively simple procedure under local anesthesia or even without anesthesia.

To reduce digestive secretions, somatostatin and its analogues are used, with anecdotal evidence of a decrease in fistula output.^[11,28]^ In addition, TPN but not EN should be used at this fistula tract formation stage even if bowel function has been restored because TPN can reduce the secretion of digestive juices more than EN.^[11]^

In a literature report, 2 negative pressure drainage techniques were described. One technique is continuous intraluminal infusion and aspiration,^[29]^ which was reported in 1984 and yielded a spontaneous closure rate of 65% but a recurrence rate of 13% and a mortality rate of 22%. In our opinion, despite the spontaneous closure rate of 65%, this approach has 2 disadvantages. First, the drainage tubes consist of 3 silicone tubes simply secured together by nylon ties, including 1 for air entry, 1 for infusion, and 1 for suction. Due to the simple trussed tubes and the casing structure, the suction tube may be easily plugged by the loose and edematous intestinal mucosa. Second, intraluminal drainage must be performed during surgery, which is difficult and fails to comply with the damage control principle. The other technique is negative pressure wound therapy for enterocutaneous fistulas,^[23,30]^ which develop from the treatment of wounds in skin or soft tissue. Therefore, most negative pressure wound therapy techniques are described in the treatment of enteroatmospheric fistulas, which are pathological communications between the intestinal lumen and the surface of an open abdominal wound. If the location of the fistula is deep in the peritoneal cavity, negative pressure wound therapy may not apply. Despite being reported to treat duodenal fistulas, this approach is also performed during complex laparotomy in which multiple tubes are positioned near the duodenal hole and the wound is filled with polyurethane sponge.^[23]^ In our opinion, the placement of multiple tubes and the fullness of a polyurethane sponge are not necessary because the infected cavity constricts slowly due to intra-abdominal pressure and finally forms a controlled tract when one drainage tube is placed in the correct position.

After identification of fistula tract formation by fistulography, promotion of healing of the fistula tract is the second treatment target. Because of the stable and controlled fistula tract, the abdominal infection can resolve and will not develop into anything more severe. Thus, at this treatment stage, somatostatin should be reduced and gradually stopped. Loperamide hydrochloride, a μ-opioid receptor agonist that can activate the μ-opioid receptor in the bowel wall to reduce intestinal movement and secretion, can be administered if the fistula output remains high after somatostatin is stopped. Moreover, EN or oral feeding with a liquid diet should be gradually administered to completely replace TPN after intestinal function recovery. Compared with TPN, EN, or oral feeding is more effective at restoring intestinal barrier function and preventing bacterial translocation. Another benefit is that EN is cheaper than TPN, which reduces the economic burden of fistula patients. If the output increases in the small bowel fistula after EN or oral feeding, oral loperamide can effectively reduce intestinal secretion. In addition, the administration of a refined elemental diet with low acidity and low fat via the jejunal route evokes no stimulation of gastric, biliary, or pancreatic secretions.

To facilitate rapid fistula tract closure, somatotropin analogues (recombinant human growth hormone) were administered in this series and demonstrated excellent efficacy. Growth hormone is a peptide hormone that stimulates the proliferation and differentiation of many types of cells and granulation tissue formation. In our opinion, recombinant human growth hormone should be used after somatostatin discontinuation and intestinal function recovery because somatotropin and somatostatin present antagonistic effects, and the fistula cannot be closed with intestinal tract obstruction.

An interesting phenomenon in this series is that the healing time of the fistula in cases 1 and 2 was remarkably shorter than that in case 3. This phenomenon may reveal a rule that despite the difficulty of placing the drainage tube, with a deep location in the abdominal cavity, fistulas heal more easily. The reasons are as follows. With a deep location in the abdominal cavity, the triple-cavity tube is surrounded by peritoneal tissues that have a strong capability for fibrin exudation and adhesion; thus, a controlled fistula tract is easy to create. The other reason is that when the drainage tube is gradually pulled out, the fistula tract can rapidly be compacted under abdominal pressure, which can provide an opportunity for mutual adhesion closure of the tract wall. In contrast, the fistula tract with a shorter length in case 3 was surrounded by subcutaneous fat tissue, which has a weak capability for fibrin exudation, resulting in a longer healing time.

The results of this study are subject to some limitations. First, this study has a limitation due to its small numbers of cases. Second, the technical solution proposed here is not adequate for every condition of enterocutaneous fistulas. It is not suitable for treatment of enterocutaneous fistulas that cannot create an effective drainage tract.

## 4 Conclusion

In conclusion, the management of postoperative high-output enterocutaneous fistula is certainly the most complex aspect, requiring a reasonable treatment strategy to achieve cure. Based on the damage control principle, management should prioritize nonsurgical treatment with a multidisciplinary approach. The strategy involving continuous irrigation and suction with triple-cavity drainage tubes in combination with sequential somatostatin–somatotropin administration may be a safe and effective alternative treatment and a more practical method that is easy to execute to manage this problem. Long-term studies, involving more patients, are still necessary to confirm this suggestion.

## Author contributions

**Conceptualization:** Yuning Cao, Daogui Yang.

**Data curation:** Xiangheng Kong, Xiangyang Zhang.

**Formal analysis:** Xiangyang Zhang.

**Investigation:** Daogui Yang.

**Methodology:** Yuning Cao.

**Project administration:** Daogui Yang.

**Software:** Xiangyang Zhang.

**Supervision:** Xiangyang Zhang.

**Validation:** Xiangyang Zhang.

**Writing – original draft:** Xiangheng Kong.

**Writing – review & editing:** Xiangheng Kong.
